# Supplementary figures and images for: Therapeutic Target Discovery for Multiple Myeloma: Identifying Druggable Genes via Mendelian Randomization
Source: Biomedicines. 2025 Apr 5;13(4):885. doi: 10.3390/biomedicines13040885 (PMC12024999; doi:10.3390/biomedicines13040885)

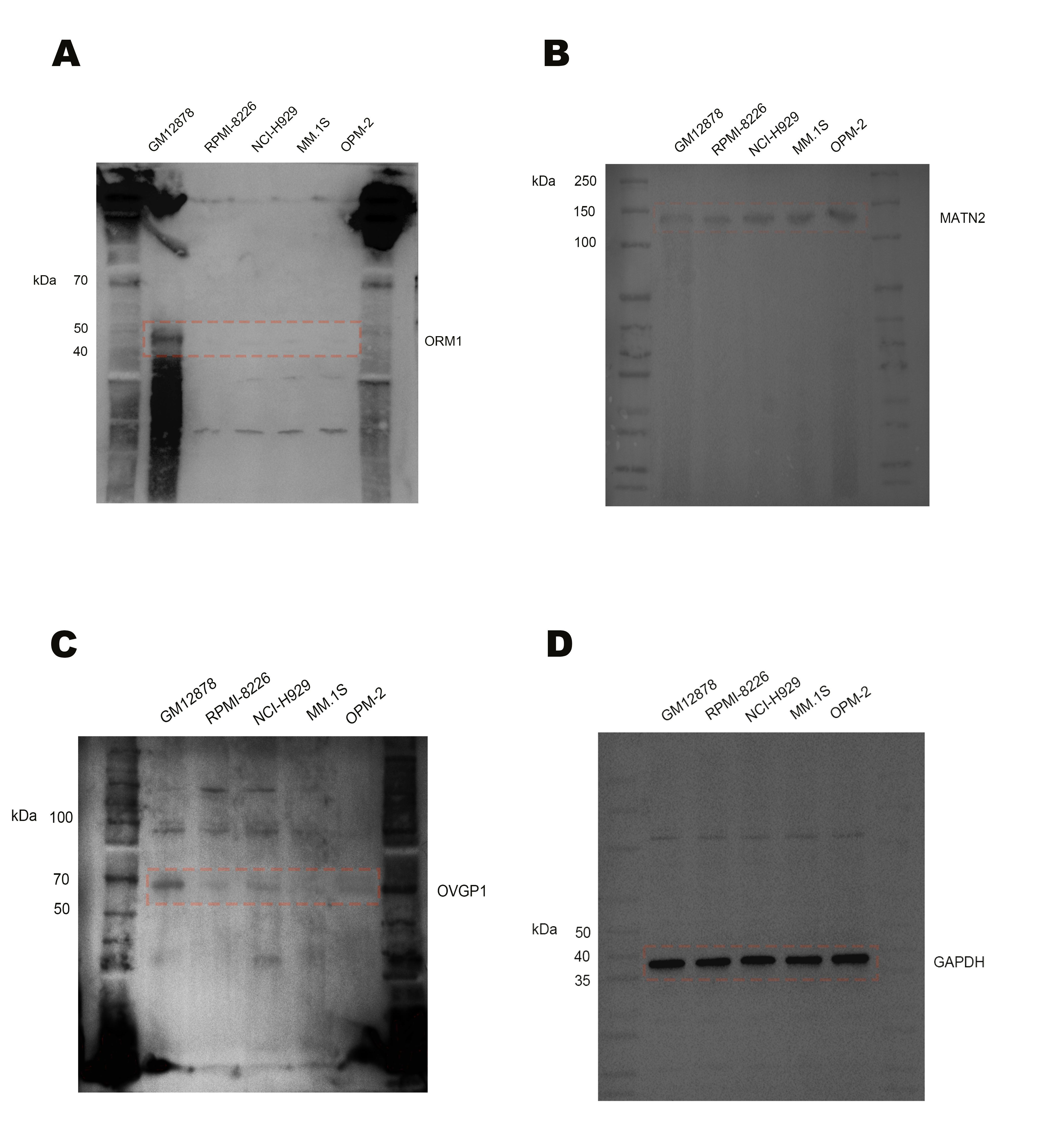

Supplement: Supplementary file 1 [file biomedicines-13-00885-s001.zip › Figure S1.png]

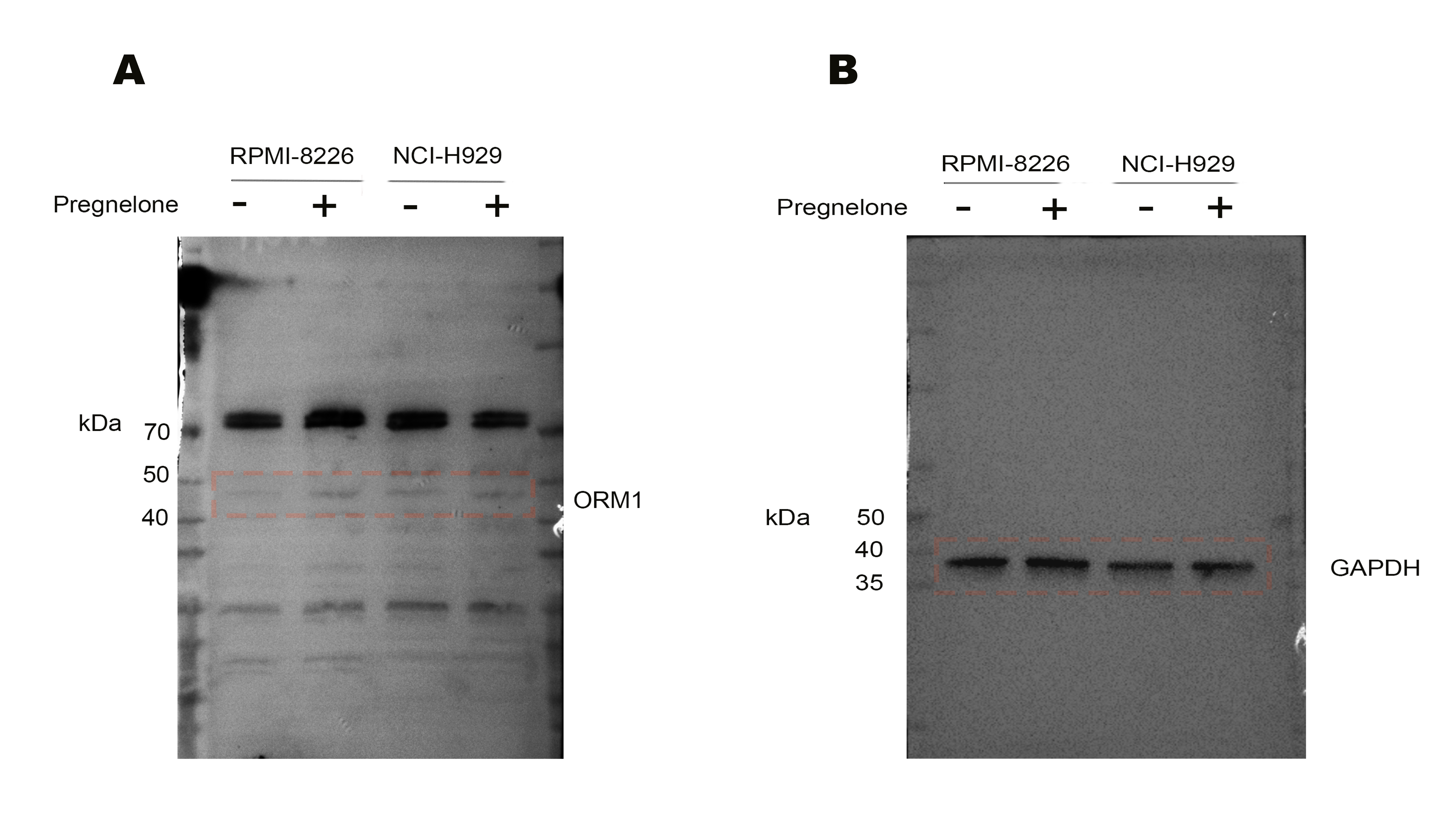

Supplement: Supplementary file 1 [file biomedicines-13-00885-s001.zip › Figure S2.png]

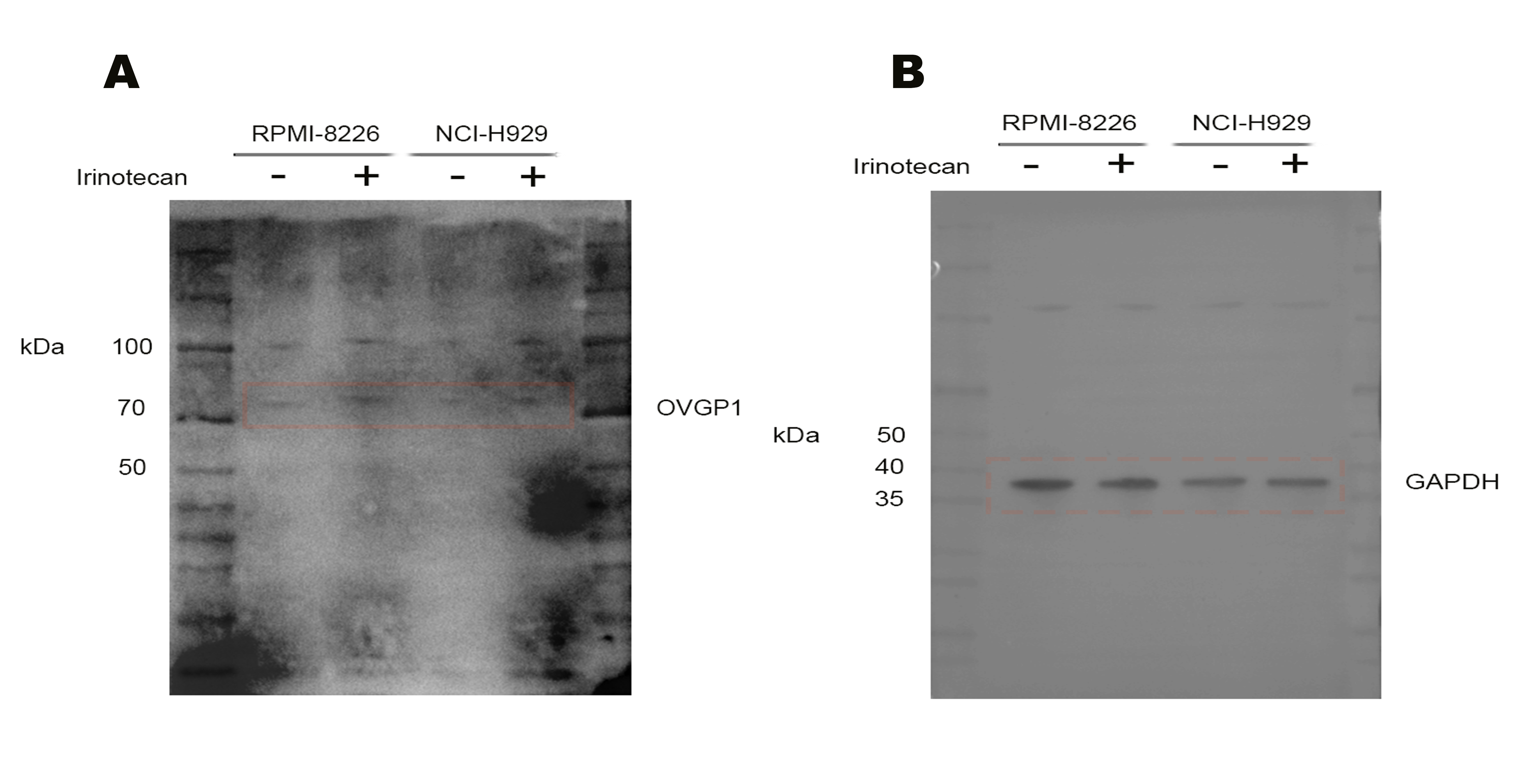

Supplement: Supplementary file 1 [file biomedicines-13-00885-s001.zip › Figure S3.png]
